# Improving Adult Vaccination Status in the United States

**DOI:** 10.3390/healthcare9111411

**Published:** 2021-10-21

**Authors:** R. Burciaga Valdez, Korazon S. Romero

**Affiliations:** 1 Department of Family & Community Medicine and Economics, University of New Mexico, Albuquerque, NM 87131-0001, USA; 2 School of Medicine, University of New Mexico, Albuquerque, NM 87131-0001, USA; KoSRomero@salud.unm.edu

**Keywords:** flu immunizations, COVID-19 immunizations, infection control, adult immunization, multiple injections

## Abstract

Adult immunization practices leave much to be desired. Misinformation has increased mistrust. As a result, Latino and African American populations have low rates of annual flu vaccinations and, during the COVID-19 pandemic, lag behind for COVID-19 vaccination. Historically, healthcare staff have failed to adhere to adult immunization guidelines contributing to patient infections. Healthcare staff, both clinical and non-clinical, must lead by example by making “prevention primary”. Most adults may not realize they need immunizations. We recommend the following steps to increase immunization uptake: Make adult immunization a standard of patient care as we do for children. Assess immunization status at every clinical opportunity. Strongly recommend vaccinations needed. Administer needed vaccinations, multiple if warranted. Document vaccines received by your patient. Participate in your state’s immunization registry and work with community organizations that can help make adult immunization the norm.

## 1. Introduction

For years, we have been concerned about the low take-up of influenza vaccines by African American and Latino adults [1]. The low take-up in these communities, but also in other communities, has been attributed to members expressing: (1) the attitude: “healthy, so don’t need it”, (2) safety concerns, (3) religious or philosophical beliefs, (4) out-of-pocket cost concerns, and (5) misinformation/conspiracy ideas. These same concerns are expressed today, hindering COVID-19 vaccinations across a larger cross-section of the nation. Reviewing what we know about flu vaccinations holds lessons for moving forward with future adult vaccination efforts, including increasing uptake of annual influenza vaccinations.

Influenza most often leads to mild illness. As a result, people have come to believe that influenza is not a severe disease. Thus, healthy individuals feel that influenza may be no more concerning than the common cold. Therefore, they see no urgency or significant concern about an infection in the short or long term.

The seasonal flu causes millions of illnesses, hundreds of thousands of hospitalizations, and tens of thousands of deaths every year in the United States [2]. It also costs USD 10.4 billion annually in direct health care costs and results in USD 16.3 billion of lost earnings [3]. While vaccination is the most effective tool to combat influenza infection, vaccination rates remain well below the Office of Disease Prevention and Health Promotion targets. The factors that affect whether people choose to vaccinate vary from community to community. However, they are related to barriers within the current health care system and notions about the risks and benefits of vaccination.

The United States has one of the highest costs of healthcare in the world [4]. Thus, people experience economic barriers to healthcare as a significant problem. A common reason that individuals are not vaccinated is due to out-of-pocket costs, which is especially true for minority communities. The lack of health insurance coverage, particularly by African American and Hispanic populations, creates financial barriers to medical care, including preventive care services [5]. A key predictor of influenza vaccination in the Hispanic community is healthcare coverage, which illustrates the importance of ensuring financial access to health care [6]. Vaccinations, including annual influenza vaccines, often require out-of-pocket expenses even when people have health insurance coverage in the form of co-payments or the full cost of service in pharmacies, even with improvements in coverage of preventive services under the Affordable Care Act [7] and the Medicare program.

Some communities operate public health vaccination clinics that may offer low or no-cost vaccinations. However, these clinics often pose other barriers to services, including transportation and hours of operation. Other economic barriers include the time taken away from work, which can be difficult for low-income families to overcome. Making low-cost vaccination available, utilizing regular office visits for vaccination, setting up mobile vaccination clinics, and offering flexible hours for public health vaccination clinics may aid in increasing vaccination levels [8].

In addition to cost, language barriers limit access to care that may contribute to low vaccination levels. Census data indicate that Hispanics are the fastest-growing ethnic group in the U.S. and 13.5% of the population speaks Spanish at home. However, the lack of Spanish-speaking providers poses a structural barrier to accessing and using health care [9]. Spanish speakers have vaccination rates that are significantly lower than Hispanics with English language preference. Creating a healthcare environment reflective of the patients served can help diminish language and cultural perceptions as barriers to care. Hiring multilingual and multicultural clinical staff is essential for healthcare organizations that wish to serve Hispanic and other communities that prefer to speak languages other than English and see the world through a different cultural lens.

The success of vaccination over the last 50 years has resulted in new generations of adults never experiencing the ravages of vaccine-preventable diseases. Smallpox eradication and the reduction of polio and measles globally means that many adults have not experienced the illness, disabilities, or death that these diseases inflicted. Increased skepticism regarding vaccination has slowed polio and measles eradication globally [10]. Some critics view the injection of a denatured infectious agent as unsafe. Others fear the possibility of vaccine adverse reactions more than the illness, even from devastating or deadly diseases such as we are experiencing with COVID-19. More recently, objections to vaccination on religious or personal philosophical beliefs have surfaced. Yet, no major religion objects to vaccination. Religious leaders argue that vaccinations serve the greater good of the community and the welfare of others [11]. Many religious leaders argue it is a moral obligation to get vaccinated against infectious and contagious diseases.

Of course, language and culture are not the only aspects of the community that health care providers must consider. The lack of trust between health care providers and the Latino and African American community is a fundamental weakness in the system. There is a strong relationship between mistrust of physicians and vaccine hesitancy [12]. In general, the American public and minority communities have become increasingly skeptical of medical providers [13]. This mistrust stems from discrimination documented for over a century and studied as “health disparities” since the early 1990s [14]. To address this trust barrier, health care providers must acknowledge that the skepticism is rational due to a history of unethical experimentation in these communities and educate themselves in ways to speak to patients, so they are heard and feel safe [15,16].

In addition to mistrust, and possibly stemming from this mistrust, many misconceptions about vaccinations have also surfaced. Many people believe that they acquire influenza from the influenza vaccine. This misconception likely comes from two situations: people mistake minor side effects of the vaccine for the illness, or people catch the flu soon after vaccination before their body has a chance to ramp up an adequate immune response. The flu shot is made from inactivated influenza virus or with a single protein from the influenza virus. The nasal spray contains live viruses that have been weakened to the point that they cannot cause illness. Having these aspects of the virus introduced to the body allows the immune system to learn and recognize the virus and create antibodies that fight off any future infections. This process takes about two weeks, which means that while our bodies work to create an immune response to the influenza virus, we may encounter and contract the virus [17].

Side effects that occur within 1–2 days can also lead people to believe they are sick. While the most common side effects of the flu shot are injection site soreness, redness, and tenderness, some people do report low-grade fever, headache, and muscle aches. These reactions are typically not as severe as the influenza illness, occur soon after vaccination, and last only 1–2 days [17]. There have also been reports of syncope after receiving the flu shot. However, this is a recurrent finding with many medical procedures, and nearly all vaccinations have reports of syncope. Therefore, events occurring with vaccination may lead to syncope, not the flu vaccine itself [18]. These side effects can also contribute to the concern for vaccine safety. Some patients remain uneasy regarding vaccine safety despite the extensive monitoring of vaccines. However, the influenza vaccine has extensive research supporting its safety, and side effects are generally mild.

A rare association with influenza vaccination is Guillain–Barre syndrome (GBS), estimated to affect one individual per million people vaccinated. Studies assessing this rare risk find no association between vaccination and GBS. GBS has a stronger association with influenza illness than influenza vaccination [18].

The misinterpretation of side effects and illness after vaccination where people are not yet protected has also led to the misconception that the flu shot is less effective. They are likely to question the effectiveness of the vaccination if they become ill. Therefore, people must understand the time it takes for the immune system to recognize and fight off the influenza virus and the potential side effects of a flu shot versus the actual illness. These normal biological circumstances do not diminish the vaccine’s efficacy to prevent moderate or severe disease and death.

Other factors contributing to ineffective flu vaccination perceptions include influenza-like illnesses, different influenza strains, and varying immune responses among the population. While it is possible to contract the flu before the vaccine has time to work, it is also likely that people become ill from a pathogen unrelated to influenza. Some symptoms may overlap with a flu illness, leading people to believe it is the flu when it is not. The influenza vaccine offers no protection against other pathogens. The vaccine also has no association with an increased likelihood of influenza-like illnesses [19]. Thus, it becomes incumbent among health care professionals to educate patients about influenza and influenza-like disease.

Differing strains of the influenza virus and differing immune responses to vaccination also make it difficult to convince people of the efficacy of vaccination. Different strains of the influenza virus circulate globally, so matching the annual vaccine components to the strains people will encounter in their area has been challenging for some years. In years with a good match, the vaccine decreases the risk of going to the doctor by 40–60% and reduces flu-related hospitalizations by about half [20].

There are different responses to the influenza vaccine even when it is an excellent match to the circulating virus strains, depending on age and health. Individuals with weakened immune systems may have a diminished capacity to create an immune response to the flu, even with vaccination. Adults 65 years and older and adults with chronic health conditions remain the highest risk groups of severe complications related to influenza virus infection [21]. The influenza vaccine is associated with lower rates of major adverse cardiovascular events [22] and decreased hospitalizations in those with chronic lung diseases [23]. In patients with diabetes, hospitalizations are reduced by 41% [24]. The influenza vaccination decreases the risk of hospitalization, intensive care unit admissions, and even death [25,26]. Among the elderly and those with chronic health conditions, vaccination is crucial due to its protection against hospitalization and death.

While it is essential for those at high risk to be vaccinated, it is also crucial for healthy individuals to be vaccinated. The flu can affect anyone, and the misconception that healthy people do not need the vaccination can increase the likelihood that people contract the virus and pass it onto others and/or become seriously ill themselves. The spread of influenza is dangerous due to the severe complications that can occur following infection. The flu can lead to complicated recoveries even among healthy adults, young children, older adults, and people with chronic health conditions. Complications range from moderate problems, such as sinus and ear infections, to more severe complications like pneumonia. The flu can also worsen preexisting health conditions leading to inflammatory responses including myocarditis, encephalitis, myositis, and rhabdomyolysis. Sepsis, a life-threatening inflammatory response to infection, can also occur because of influenza. People can overlook these complications because of the misconception that influenza is not a severe illness, which is far from the truth.

Timing is also a misconception that prevents vaccination. The optimal time to get vaccinated is as soon in the season as possible, usually in late September or October. Even if a person has contracted the flu, vaccination is recommended because there are different flu strains, and it is usually unknown which caused the infection. This year influenza vaccine contains four virus strains. Therefore vaccination, even in a recently infected person, adds protection against the other influenza strains [27]. Vaccination is also recommended through the spring months if unexpired vaccines and unvaccinated patients exist. The peak of the flu season typically occurs between December and March, but infection can occur as late as May.

While there are facts that counter misconceptions surrounding vaccination, healthcare providers have lagged in their response to facing vaccine hesitancy head-on. Vaccine hesitancy challenges doctors, scientists, advocates, and often family and friends [28]. Vaccine hesitancy remains a persistent global threat that is beginning to be better understood [29,30] because of recent work regarding COVID-19 vaccination hesitancy. However, even with the severity of the current COVID-19 pandemic, overcoming vaccination hesitancy where it exists remains a high priority for achieving control over the pandemic.

## 2. The COVID-19 Pandemic and Vaccination

The concerns surrounding vaccination have become more apparent during the COVID-19 pandemic. While there is considerable overlap behind adult vaccine hesitancy, such as the various misconceptions previously mentioned, there are also factors specific to the onset of this pandemic. The COVID-19 vaccine technologies vary from previous vaccines, such as those in use against influenza. Pfizer and Moderna vaccines use mRNA technology developed over the last few decades [31]. These vaccines allow mRNA from the SARS-CoV-2 virus spike protein to be introduced, covered by a chemical capsule. Human cells use the mRNA to make antibodies that prevent the virus from attaching to cell structures.

Other COVID-19 vaccine producers like Janssen (Johnson & Johnson) and Oxford/AstraZeneca use adenovirus technology, which was first used in the 1970s [32]. The adenovirus technology, much like mRNA technology, introduces genetic material from the SARS-CoV-2 virus spike protein. Adenovirus vaccines use common viruses such as the virus that causes the common cold (Janssen uses recombinant, replication-incompetent adenovirus type 26; AstraZeneca uses a chimpanzee common cold viral vector known as ChAdOx1), which are deactivated by removing critical parts of the virus’s DNA. These DNA pieces are replaced with segments of the SARS-CoV-2 virus spike protein. The body mounts an immune response to the spike protein components. Neither the m-RNA nor the adenovirus COVID-19 vaccine technologies use the infectious component of the virus to make the vaccine and instead use the genetic blueprint of the spike protein used by the virus to infect cell structures.

The COVID-19 vaccine is a novel vaccine created at an accelerated pace, causing fears about safety. However, the public needs to recognize that these vaccines were developed after more than a decade of research on mRNA and adenovirus vaccines. The clinical trials for these vaccines used existing networks. Further, manufacturing of the vaccines started before the completion of phase III clinical trials [33], so should they prove safe and effective, they could be available quickly. The mRNA and adenovirus technology allows for quicker production than older technologies because producing the genetic material of the COVID-19 spike protein is a faster process than making the entire COVID-19 virus needed for a live attenuated vaccine. While pharmaceutical companies accelerated the development of the COVID-19 vaccines, no corners were cut when validating the safety and efficacy of the vaccines. **The federal government eliminated the financial risks that pharmaceutical companies usually take on when creating and manufacturing a new product and delay their time to market.**

Concerns surrounding the COVID-19 vaccines are keen in the African American and Hispanic communities. Distrust of both the government and the healthcare system underlies many of these concerns. Further, COVID-19 disproportionately affected these minority groups over the last year laying bare the inequities in our society. When recommending vaccinations, it is important to recognize historical traumas, validate concerns, and work with patients to create a trusting relationship that promotes health [34]. It is also important to recognize how misinformation has eroded trust even further.

The sources expanded, and the intensity of misinformation about vaccines intensified over the last decade with the increased number of people who rely on social media for their information. A recent study illustrates that misinformation from social media raises doubts about vaccination safety and that foreign misinformation such as various Russian actors has pushed anti-vaccination messages to the West on a large scale [35]. For example, globally, we observed polio eradication prevented by misconceptions such as the false message that the vaccine causes sterility or infertility [36]. Addressing people’s fears and concerns about vaccinations directly can reassure those reluctant to “take the jab.”

While vaccine hesitancy for COVID-19 has shown a decrease over the last few months with the appearance of the Delta variant, it is vital to counter the anti-vaccination messages and myths circulating online and in the mass media. We must combat misinformation promoted by individuals and groups that reinforce misconceptions about the risks and benefits of vaccination for flu, COVID-19, and other vaccine-preventable diseases.

## 3. Recommendations to Improve Adult Vaccination Uptake

Clinical and non-clinical staff are in regular contact with patients and exposed frequently to viruses and bacteria. They are at high risk for developing vaccine-preventable diseases. They may also be a source of infection for vulnerable patients and colleagues. Adherence to the CDC’s Advisory Committee on Immunization Practices (ACIP) vaccination recommendations for health care professionals by all clinic staff is a critical infection prevention and control measure [37].

Clinic and hospital staff historically have poor vaccination coverage rates that have remained low despite repeated recommendations for annual influenza vaccination. We cannot afford to continue putting patients at risk of vaccine-preventable illnesses, especially potentially deadly diseases such as COVID-19. Vaccination of all staff benefits patients, colleagues, and community members. In addition, leading by example increases public confidence and trust regarding the efficacy and safety of adult vaccinations.

Non-clinical staff who more often reflect the community’s diversity can champion immunization by educating the public on the importance and safety of vaccinations [38]. We must adopt intellectual humility in our staff, patient, and public education efforts. We must win them over to a vaccination norm by listening carefully to their concerns, asking and answering all questions, and appealing to their communitarian values. Building trust can be frustrating as we strive to keep staff and patients from becoming defensive. We must slowly welcome them to a new place in their thinking, sometimes through many invitations to cross the threshold.

Proven strategies to increase vaccination coverage among adults are available for health care providers and institutions to implement [39]. Practices serving children have made childhood vaccination a priority. Adult practices could improve recommended ACIP vaccinations by screening for vaccine needs during regular ambulatory care contacts, emergency room visits, or during hospital discharge. Practices that treat large numbers of patients at high risk for vaccine-preventable illnesses (e.g., patients with diabetes, asthma, heart failure, and the elderly) should implement systematic monitoring of immunization status [40]. Vaccination of older adults in long-term care facilities, receiving home care, or being served by managed care organizations can occur because of standing orders or routine annual protocols, as CMS no longer requires a physician order for influenza or pneumococcal administration [41].

The ACA requires health insurance policies to provide full reimbursement for recommended vaccines with no out-of-pocket costs for patients. Amazingly, Medicare and Medicaid pay only for some ACIP-recommended vaccines. Healthcare providers and public health authorities must advocate for reimbursement for all recommended vaccines and their administration and free vaccination programs for the uninsured. Vaccinations are among the most cost-effective disease control and prevention measures available, reducing costly hospitalizations and deaths.

Low adult vaccination coverage requires more aggressive efforts by public health authorities and every medical provider to inform and diligently answer every concern about vaccinations that the public may harbor. Vaccination is not different from any other medical procedure requiring informed consent. Unfortunately, increased workflow pressures may shift the focus from a robust conversation, education, and shared decision making to merely getting a signature on a form [42,43]. Dispelling vaccine myths requires public education and shared decision making, which may include repeated screenings and discussions.

Health care providers and their staff can influence and motivate patients to be fully immunized. Many adults are not aware that they need vaccinations and rarely ask for them when they seek care [44]. It has been recognized but become even more evident during the COVID-19 pandemic that many providers and staff may also need education about adult vaccines [45]. All staff must develop the skills to counsel patients with individualized messages addressing any concerns about a vaccine, its efficacy, effectiveness, and safety and respectfully address misconceptions or myths with facts. Myths are plentiful causing fear about vaccination rather than the serious diseases they can help control.

Enlisting trusted community partners in ongoing efforts to improve adult vaccinations cannot simply be a strategy during epidemics or pandemics. Our highly fragmented delivery system requires outside assistance from community non-profit organizations and other institutions to sustain vaccination efforts through public policy advocacy, communicating the benefits of vaccinations on social media, and holding public events. For example, our annual influenza vaccination campaigns could include workplace and higher education settings to reach those who may not make regular visits to the pharmacy or health care providers. Delivery of preventive services such as vaccinations must go where the public is to be found rather than expecting the public to come to health care institutions or physician offices. Community organizations are the trusted bridge from the medical and public health authorities to the people that can make this possible. Today, building and maintaining trusted relationships with community organizations benefit efforts to address emergencies tomorrow and disease control and prevention every day. The Immunization Action Coalition (https://www.immunize.org/adult-vaccination/) (accessed on 2 September 2021) provides resources for health professionals, the public, and local coalitions to support adult vaccination efforts. Vaccinations are one of numerous measures (e.g., masking, physical distancing, improved ventilation) that reduce the risk of respiratory infections such as influenza and COVID-19 [46]. Multiple measures along with mass vaccination globally are necessary to end the current COVID-19 pandemic.

## Data Availability

Not applicable.
